# Lung cancer risk among textile workers in Lithuania

**DOI:** 10.1186/1745-6673-2-14

**Published:** 2007-11-16

**Authors:** Irena Kuzmickiene, Mecys Stukonis

**Affiliations:** 1 Institute of Oncology, Vilnius University, Lithuania

## Abstract

**Background:**

The textile industry is one of the largest employers in Lithuania. IARC monograph concludes that working in the textile manufacturing industry entails exposures that are possibly carcinogenic to humans. The purpose of this study was to investigate risk of lung cancer incidence in textile industry workers by the type of job and evaluate the relation between occupational textile dusts exposure and lung cancer risk in a cohort.

**Methods:**

Altogether 14650 textile workers were included in this retrospective study and were followed from 1978 to 2002. Lung cancer risk was analyzed using the standardized incidence ratios (SIR) calculated by the person-years method. The expected number of cases was calculated by indirect methods using Lithuanian incidence rates.

**Results:**

During the period of 25 years 70 cancer cases for male and 15 for female were identified. The SIR for male was 0.94 (95% CI PI 0.73–1.19), for female 1.36 (95% CI 0.76–2.25). The lung cancer risk for male in the cotton textile production unit was significantly lower after 10 years of employment (SIR = 0.34; 95% CI 0.12–0.73). The lung cancer risk decreased with level of exposure to textile dust (p for trends was <0.05): the SIR for the low, medium, high and very high level of cumulative exposure were 1.91 (95% CI 0.92–3.51), 1.30 (95% CI 0.52–2.69), 0.77 (95% CI 0.21–1.96), and 0.24 (95% CI 0.03–0.86) respectively.

**Conclusion:**

In our study the exposure to cotton textile dust at workplaces for male is associated with adverse lung cancer risk effects. High level of exposure to cotton dusts appears to be associated with a reduced risk of lung cancer in cotton textile workers.

## Background

The textile industry is one of the largest employers in Lithuania. The textile includes spinning, weaving, knitting and finishing all types of natural and artificial (synthetic) fibers. IARC monograph concludes that working in the textile manufacturing industry entails exposures that are possibly carcinogenic to humans [1]. During spinning, weaving and knitting operations, exposure to chemicals is generally limited. Exposure to natural organic (cotton) fibers in textile industry is usually characterized as cotton dust [2]. Numerous studies have shown that exposure to organic dust, especially that having endotoxin, results in lower rates of lung cancer [3-7]. Several recent investigations have reported decreased risks of lung cancer among workers in the cotton textile industry [8-10]. A several studies do not indicate an increased risk of cancer, rather a decreased risk [11,12]. In our previously report, we presented the cancer risk in the cotton textile workers cohort in 1979–1997 [13]. The lung cancer risk in this workers cohort was slightly increased than in the general country population. However, our study results may be the results of young age of cohort and the small of number cases. The follow up was prolonged for five years. The aim of the present study was to investigate lung cancer incidence risk in relation to the type of job and textile dust exposure.

## Methods

The retrospective cohort consisted of persons found on the payroll of cotton textile mill in Alytus. The study included all individuals who were employed in the mill for at least 1 year. Workers with less than one year of employment were excluded. The employment period for Alytus textile mill was from 1 January, 1969 trough to December 31, 2002. The date of follow up began on 1 January 1978, or the date of first employment after this date. The follow up ended on the date of death, date of emigration, or disappearance obtained from the Residents' Register Service, or on 31 December 2002, for those known to be living in Lithuania at the closing date. The vital status of the cohort members was in Lithuanian Archives Department under the Government of the Republic of Lithuania, Residents' Register Service and Migration Department under the Ministry of Interior.

The mill supplied information about name and surname, sex, date of birth, dates of employment and retirement, workshop, and task description, by abstracting the relevant information from employment records. Industrial hygiene assessment was conducted in order to the authors with past and current operations and estimate textile dust and chemical exposures in each department of the mill. Based on this information, the workers have been classified in thee groups: a) cotton textile production unit; b) cotton textile finishing unit; c) maintenance unit. The division between workers should reflect differences in the degree of lung cancer risk which are correlated with differences in working conditions.

The basic raw materials used in textile production were fibers, obtained from cotton but beginning with 1991 some other types of fibers was also processed (cotton/polyester, polyester/rayon). Since the early 1977s, the mill's laboratory has been monitoring the levels of dust and noise, as well as airborne concentrations of chemicals in the ambient air of the production rooms. The group of textile finishing departments (bleaching, dyeing, printing and other finishing) was characterized by high exposure to chemicals. The percentage of the subjects employed in that unit was 8.8%. Cotton textile production (spinning and weaving) workers have close contact with textile dust. The majority of cohort members (56%) were exposed to dust and noise. Average airborne dust concentrations found ranged from 1.10 mg/m^3 ^to 28.60 mg/m^3^; average total dust concentrations in all areas were above the threshold limit value of 1 mg/m^3^.

The cumulative exposure to total dust was calculated for every subject by combining the exposure matrix and work history, with the following equation: cumulative exposure (CE) was calculated as a product of prevalence, level and estimated duration of exposure to textile dust [14]. Cumulative exposure to total dust: (mg/m^3 ^year): ∑n_i _= 1 (C_i_*T_i_), where: C_i _= total airborne cotton dust concentration for the job and employment period obtained from the job – exposure matrix; T_i _= duration of employment (years) of subject for the job (i) from work history, it was adjusted by the number worked/day, one year in dust defined as 8 hours/day and 270 days/year. An average level of exposure to textile dust was assigned to the four quartiles: low exposure (>0 to < 8,0 mg/m^3^-yr), medium exposure (from 8,1 to 19,7 mg/m^3^-yr), high exposure (form 19,8 to 64,7 mg/m^3^-yr), very high exposure (from 64,8 to 200,6) and no exposure. Workers who have performed maintenance in the exposed areas, and other no exposed workers were used as the lowest exposed group for comparison.

Information on new cancer cases (coded according to the international classification of diseases, 9th revision (ICD-9) diagnosed from 1978–2002, was obtained from the Cancer Registry of Institute of Oncology, Vilnius University. Cancer cases were identified by record linkage to the cancer register.

To calculate the standardized lung cancer incidence ratios, we divided the observed numbers of cases by the corresponding expected numbers. The expected number of cases was calculated by indirect method using Lithuanian incidence. The person years at risk for each worker was calculated from 1 January 1978 or the date of first employment after this date until 31 December 2002, date of death or residence abroad. The main conclusions are based only on statistically significant SIRs. We also employed a person-years method, using Poisson regression, to calculate standardized incidence ratios (SIRs) to compare risks in the different exposure subgroups, with risks in the comparison subcohort. We used the Mantel-Haenszel chi-square test for trend. We defined the exact 95% confidence interval for each ratio on the assumption that the number of observed cases followed a Poisson distribution [15,16]. For categories with fewer than 20 observed cancer cases, exact 95% Poisson confidence intervals were used [17]. A p value < 0.05 was considered significant.

## Results

A total of 14650 subjects (5495 male and 9155 female) were identified and proved the vital status. 13252 subjects (90.5%) were classified as alive, 1009 (6.9%) as deceased, 82 (0.6%) had emigrated and 307 (2.1%) as having an undetermined vital status. The number of person years at risk was 84196 for male and 167607 for female. Table 1 gives further characteristics of the distributions of the included subjects such as age at of beginning of study, age of end of study, length of service, job category, and number of lost to follow-up.

**Table 1:** Characteristics of the cohort of Alytus textile mill workers from 1978 through 2002.

*Characteristic*	*Male*	*Female*	*Total*
Number of workers	5495	9155	14650
Person years	84196	167607	372018
Mean age of beginning of study (SD)*	28.9 (8.6)	31.1 (9.1)	28.8 (8.2)
Mean age of end of study (SD)	47.9 (7.9)	47.0 (6.3)	47.4 (7.1)
Mean length (years) of service, (SD)	12.0 (5.1)	10.1 (5.8)	12.3 (5.9)
Number of lost to follow-up (%)	128 (2.3)	179 (1.9)	307 (2.1)

*SD = standard deviation.

A total of 70 lung cancer were observed in male, yielding a non-significantly decreased risk (SIR = 0.94; 95% CI 0.73–1.19). In contrast, for female the lung cancers were more common than expected (15 cases, SIR = 1.36; 95% CI 0.76–2.25) (table 2). For male in the cotton textile production unit SIR for lung cancer was 0.95 (95% CI 0.60–1.41) and for female 1.50 (95% CI 0.68 – 2.84). No excess risk was found for textile finishers, but only for female cotton textile finishing workers SIR no significantly increased to 2.17 (95% CI 0.59 – 5.67, based on 4 cases). The SIRs of lung cancer incidence among maintenance workers were similar to those in the general population of the Lithuania.

**Table 2:** Standardized incidence ratios (SIR) for lung cancer by sex of Alytus textile mill workers from 1978 through 2002.

	*Male*			*Female*		
	
*Unit*	*Obs*	*SIR*	*95% CI*	*Obs*	*SIR*	*95% CI*
Cotton textile production	23	0.95	0.60–1.41	9	1.50	0.68–2.84
Cotton textile finishing	5	0.61	0.20–1.43	4	2.17	0.59–5.67
Maintenance	42	1.01	0.73–1.36	2	0.92	0.11–3.33
Total	70	0.94	0.73–1.19	15	1.36	0.76–2.25

Table 3 present standardized incidence ratios (SIR) for lung cancer by sex and units in ≥10 years of length of employment. Lung cancer was found more decreased in male textile production workers were employed 10 years or more (total 25 cases, SIR = 0.89; 95% CI 0.57–1.31). The lung cancer risk for female in this experience group was approximately the same as the general country population (4 cases, SIR = 1.27, 95% CI 0.34–3.24).

**Table 3:** Standardized incidence ratios (SIR) for lung cancer by sex and units for employment at least 10 years.

	Male			Female		
	
Unit	*Obs*	*SIR*	*95% CI*	*Obs*	*SIR*	*95% CI*
Cotton textile production	6	0.62	0.23–1.34	3	1.96	0.40–5.73
Cotton textile finishing	0	-	0.00–0.88	1	1.22	0.25–5.56
Maintenance	19	1.33	0.80–2.08	0	-	-

**Total**	25	0.89	0.57–1.31	4	1.27	0.34–3.24

Cancer risk by cumulative exposure categories to the textile dust among male cotton textile production workers were compared with maintenance group (Table 4). The significantly decreased risk for male employment in very high exposure cotton textile dust was observed (SIR = 0.24; 95% CI 0.03–0.86 and RR = 0.24; 95% CI 0.03–0.91). The significant inverse dose-response trend of SIR found between risks of lung cancer for male (p < 0.01). Compared with female who have never been exposed to the cotton dust, female textile production workers have 62% increase risk. However, lung cancer risk decreased with increasing cumulative cotton textile dust exposure (SIR in the quartiles of 2.38, 2.17, 1.28, and 0.55).

**Table 4:** Standardized incidence ratios (SIR) and rate ratio (RR) trends for lung cancer among cotton textile production unit workers by dust exposure category.

Exposure category	Obs	SIR	95% CI	RR	95% CI	χ^2 ^test for trend
***Male***						
None* (N = 2511)	42	1.01	0.73–1.36	1 (reference)		χ^2 ^= 9.68;p = 0.02
Low (N = 656)	10	1.91	0.92–3.51	1.89	0.85–3.85	
Medium (N = 481)	7	1.30	0.52–2.69	1.29	0.49–2.91	
High (N = 529)	4	0.77	0.21–1.96	0.76	0.20–2.10	
Very high (N = 574)	2	0.24	0.03–0.86	0.24	0.03–0.91	
***Female***						
None (N = 2140)	2	0.92	0.11–3.30	1 (reference)		χ^2 ^= 4.68;p = 0.20
Low (N = 1350)	3	2.38	0.49–6.91	2.58	0.30–30.9	
Medium (N = 1410)	3	2.17	0.45–6.35	2.36	0.27–28.2	
High (N = 1371)	2	1.28	0.16–4.63	1.39	0.10–19.2	
Very high (N = 1910)	1	0.55	0.01–3.08	0.60	0.01–11.5	

None * – using the maintenance employment group as the reference category.

## Discussion

Observed lung cancer incidence in the cohort was compared with expected incidence, calculated on the basis of the incidence rates of the general population in Lithuania. The SIRs demonstrated a relatively low occurrence of lung cancer for male workers at textile manufacturing mill. The standardized lung cancer incidence ratios (SIR) were higher for female than for male. There were impossible for definite conclusions because of the small number of cases among female. Many authors have shown that exposure to organic dust, especially hat having endotoxin, results in lowing rates of lung cancer [17-19]. The paper by Laakkonen et al. [20] reported a lower than expected rate of lung cancer in textile workers for male and female. The data in this study, as has been previously reported, suggest that a dose-response relationship exists for increasing cotton textile dust and lowered lung cancer rates. Our data showed that cumulative exposure to cotton textile dust was inversely related to risks of lung cancer (p for trend = 0.01) for male. A decreased risk of lung cancer has been consistently reported among different populations exposed to bacterial endotoxin, in particular, textile workers [21]. According Astrakianakis G., et al., cumulative exposure to endotoxin was strongly and inversely associated with lung risk [22]. It has been postulated that bacterial endotoxins through immunological mechanisms can be protective against lung cancer. Considering our suggestion were performed investigations of systemic immunity in women working in the Alytus textile mill in 1998–2000. This study showed that the immune functions of the female in the weaving workshop were both suppressed and stimulated: the indices of CD4^+ ^lymphocytes, leucocytes sensibilisation to enterobacterial common antigen were by 5–30% lower and CD8^+ ^and lymphocytes by 20% higher in comparison with the same indices of control group female. The population level of natural antibodies to enterobacterial common antigen, CD4^+ ^and lymphocytes, CD4^+^/CD8^+ ^indices were by 5–17 higher [23]. Other factors such as smoking habits influences on lung cancer must also be considered as potential bias. Although data on smoking habits were only available in some case control studies, the adjustment for cigarette smoking made little difference to the findings [8]. Recently, a study of female Chinese textile workers, whom few smoked, found of reduced lung cancer [24]. In our studies in progress (still in published) showed that 69% male and 20% females of textile production unit workers, and 66% males and 17.4% females of comparison group at least one year the tobacco smoked every day. The main results cannot be explained by smoking habits and/or implications of social status, i.e., could be in textile professional occupation influence.

The healthy-worker effect may be one of the factors of influence for workers in industry that have a lower incidence patterns than those of the population. May be there are gender differences in the healthy worker effect [25]. Possible gender-specific vulnerabilities are a further aspect that should also be considered. The study also has several limitations. The analysis was limited by the small number of observations. Study limitations include the lack of detailed exposure records over time and the absence of individual smoking histories. In view of the large number of exposures considered, we believe any conclusions based on our findings should be drawn with caution. A limitation of the study is the lack of information on histological type of lung cancer. This limits our ability to capture histology-specific lung cancer risk by textile dust exposure. Our results confirm the lower risk of lung cancer in the cotton textile production compared with that in the general. Current scientific evidence suggests that a small percentage of cancers could be related to exposure to endotoxins in the textile workplace [22,24,26]. More importantly, exposure to these cancer-causing substances may be preventable.

## Conclusion

In our study the exposure to cotton textile dust at workplaces for male is associated with adverse lung cancer risk effects. High level of exposure to cotton dusts appears to be associated with a reduced risk of lung cancer in cotton textile workers.

## Competing interests

The author(s) declare that they have no competing interests.

## Authors' contributions

IK and MS were responsible for the study design and writing of the manuscript. IK carried out the data acquisition and analyses. Both authors read and approved the final manuscript.

## References

[B1] International Agency for Research on Cancer (1990). Monographs on the evaluation of carcinogenic risk to humans. Some flame retardants and textile chemicals and exposures in the textile manufacturing Industry.

[B2] Neefus JD, Cralley LV, Cralley LJ (1982). Textile Industrial Processes. Industrial Hygiene Aspects of Plant Operations.

[B3] Enterline PE, Sykora JL, Keleti G, Lange JH (1985). Endotoxin, cotton dust and cancer. Lancet.

[B4] Mastrangelo G, Grange JM, Fadda E, Fedeli U, Buja A, Lange JH (2004). An exposure-dependent reduction of lung cancer risk in dairy farmers: a nested case-referent study. Indoor Built Environ.

[B5] Lange JH, Mastrangelo G, Fedeli U, Rylander R, Lee E (2003). Endotoxin exposure and lung cancer mortality by type of farming: is there a hidden dose-response relationship?. Ann Agric Environ Med.

[B6] Mastrangelo G, Grange JM, Fadda E, Fedeli U, Buja A, Lange JH (2005). Lung cancer risk: effect of dairy farming and the consequence of removing that occupational exposure. Am J Epid.

[B7] Lange JH, Rylander R, Fedeli U, Mastrangelo G (2003). Extension of the hygiene hypothesis to the association of occupational endotoxin exposure with lower lung cancer risk. J Allergy Clin Immunol.

[B8] Astrakianakis G, Seixas N, Camp J, Ray R, Gao DL, Wernli K, Thomas DB, Checkoway H (2005). Reduced lung cancer risk associated with cotton dust exposure in female textile workers in Shanghai, China. Am J Epid.

[B9] Hodgson JT, Jones RD (1990). Mortality of workers in the British cotton industry in 1968–1984. Scand J Work Environ Health.

[B10] Su W, Chen YH, Liou SH, Wu CP (2004). Meta-analysis of standard mortality ratio in cotton textile workers. Europ J Epid.

[B11] Abrams K, Fenty J, Jones DR, Levy LS, Rushton L, Sutton AJ (2004). Systematic review and meta-analysis of mortality and cancer incidence among workers in the textiles, fibers, and fabrics sector of the chemical industry [abstract]. Occup Environ Med.

[B12] Levin LI, Gao YT, Blot WJ, Zheng W, Fraumeni JF (1987). Decreased risk of lung cancer in the cotton textile industry of Shanghai. Cancer Res.

[B13] Kuzmickiene I, Didžiapetris R, Stukonis M (2004). Cancer incidence in the workers cohort of textile manufacturing factory in Alytus (Lithuania). J Occup Environ Med.

[B14] Stewart PA, Herrick RF (1991). Issues in performing retrospective exposure assessment. Appl Occup Environ Hyg.

[B15] Breslow NE, Day NE (1987). The Design and Analysis of Cohort Studies. Statistical Methods in Cancer Research.

[B16] Esteve J, Benhamou E, Raymond L (1994). Descriptive Epidemiology. Statistical methods in cancer research.

[B17] Altman DG, Gore SM, Gardner MJ, Pocock SJ, Gardner MJ, Altman DG (1989). Statistical guidelines for contributors to medical journals. Statistics with confidence.

[B18] Michie HR, Manogue KR, Spriggs DR, Revhaug A, O'Dwyer S, Dinarello CA, Cerami A, Wolff SM, Wilmore DW (1988). Detection of circulating tumour necrosis factor after end toxin administration. N Engl J Med.

[B19] Rylander R (1990). Environmental exposures with decreased risk for lung cancer. Int J Epid.

[B20] Laakkonen A, Kyyronen P, Kauppinen T, Pukkala E (2006). Occupational exposure to eight organic dusts and respiratory cancer. J Occup Environ Med.

[B21] Mastrangelo G, Fedeli U, Fadda E, Milan G, Lange JH (2002). Epidemiologic evidence of cancer risk in textile industry workers: a review and update. Tox Ind Health.

[B22] Astrakianakis G, Seixas NS, Ray R, Camp JE, Gao DL, Feng Z, Li W, Wernli KJ, Fitzgibbons ED, Thomas DB, Checkoway (2007). Lung Cancer Risk Among Female Textile Workers Exposed to Endotoxin. J Natl Cancer Inst.

[B23] Kristaponiene A, Kemekliene R, Kazbariene B, Lokiene R, Monceviciute-Eringiene E, Monceviciute-Eringiene E (2001). Endotoxin and cancer prevention. Problem of cancer prevention (Vezio profilaktikos problemos).

[B24] Zahm SH, Blair A (2003). Occupational cancer among female: where have we been and where are we going?. Am J Ind Med.

[B25] Liebers V, Bruning T, Raulf-Heimsoth M (2006). Occupational endotoxin-exposure and possible health effects on humans. Am J Ind Med.

[B26] Boffetta P (2007). Endotoxins in Lung Cancer Prevention. J Natl Cancer Inst.

